# Disparate regulation of IMD signaling drives sex differences in infection pathology in *Drosophila melanogaster*

**DOI:** 10.1073/pnas.2026554118

**Published:** 2021-08-02

**Authors:** Crystal M. Vincent, Marc S. Dionne

**Affiliations:** ^a^MRC Centre for Molecular Bacteriology and Infection, Imperial College London, London SW7 2AZ, United Kingdom;; ^b^Department of Life Sciences, Imperial College London, London SW7 2AZ, United Kingdom

**Keywords:** *Drosophila melanogaster*, IMD, PGRP-LB, infection, sexual dimorphism

## Abstract

Male and female animals exhibit differences in infection outcomes. One possible source of sexually dimorphic immunity is the sex-specific costs of immune activity or pathology, but little is known about the independent effects of immune- versus microbe-induced pathology and whether these may differ for the sexes. Here, by measuring metabolic and physiological outputs in *Drosophila melanogaster* with wild-type and mutant immune responses, we test whether the sexes are differentially impacted by these various sources of pathology and identify a critical regulator of this difference. We find that the sexes exhibit differential immune activity but similar bacteria-derived metabolic pathology. We show that female-specific immune-inducible expression of *PGRP-LB*, a negative regulator of the immune deficiency (IMD) pathway, enables females to reduce immune activity in response to reductions in bacterial numbers. In the absence of *PGRP-LB*, females are more resistant to infection, confirming the functional importance of this regulation and suggesting that female-biased immune restriction comes at a cost.

## Significance

Sex differences in infection outcome are a widely observed phenomenon. While it is known that biological sex can influence an animal’s response to infection, the mechanisms through which these differences emerge are less clear. Here, we describe a mechanism through which heightened regulation of the IMD signaling pathway by female—but not male—*Drosophila melanogaster* reduces the cost of immune activity at the expense of resistance to bacterial infection. Through the masculinization of the main organ responsible for antimicrobial peptide activity in the fly (fat body), this work demonstrates that this heightened immune regulation is mediated by sex-determining pathways.

Biological sex can influence an animal’s response to infection, with females often mounting stronger innate and adaptive immune responses compared to males. Across multiple taxa, the sexes exhibit differing incidences of infection, pathogen loads, pathogen-derived virulence, and immune efficacy (1–8). In humans, the greater responsiveness of the female immune response can confer rapid pathogen clearance, reduced mortality rates, and greater efficacy of vaccines; however, it also thought to be responsible for the increased incidence of inflammatory and autoimmune disease in women (3, 9, 10). Thus, females appear to trade-off the rapid and efficient clearance of foreign bodies, with the risk of doing self-harm, either due to autoimmunity or immunopathology. Consequently, sex-specific infection outcomes could be driven by differences between the sexes in the risks of autoimmunity, immunopathology, virulence (pathogen-induced harm), or trade-offs between immunity and other important traits.

The origins of infection-induced pathology and the mechanisms employed by hosts to limit pathology are key issues in understanding this difference between the sexes. Infection pathology can result from direct interactions between host and pathogen or can be driven indirectly. Direct pathology is caused by the pathogen itself and its products and can be produced by many effects; pathogen- or pathogen effector–driven damage to host tissue (11, 12) is the most obvious of these, but other direct pathological processes include competition with the host for access to resources (13–15). Indirect pathology, in contrast, is caused not by the pathogen itself but by some aspect of the host response to the pathogen and is most often conceived as pathology caused by immune effectors; other indirect pathologies come in the form of immune trade-offs, where immune activation leads to the reallocation of host resources from other processes, such as longevity, reproduction, competitive ability, and development (5, 16–23).

Differences in infection outcomes between hosts can result from differences in the ability of the host to clear the pathogen (“resistance” mechanisms) or from differences in sensitivity to direct or indirect pathology (“tolerance” mechanisms). In any given infection, the survival and continued health of the host will be the product of a complex interaction of host and pathogen genotype as well as other factors. It is unclear whether the well-documented effects of host sex on infection outcome in general primarily originate in changes in resistance to the infectious agent or in tolerance of direct or indirect pathology.

To distinguish these effects, we used the fruit fly *Drosophila melanogaster* and consider the response of *w*^1118^ control and immunocompromised flies to infection with the bacterium *Escherichia coli.* Unlike mammals, *D. melanogaster* lacks an adaptive immune response, instead, flies have a well-developed innate immune response consisting of both cellular and humoral components. The humoral response of *D. melanogaster* involves the inducible production of circulating factors—primarily antimicrobial peptides (AMPs)—that are directly microbicidal. Though infection with *E. coli* is nonlethal and efficiently controlled by the immune response of *w*^1118^ flies, *E. coli* infection cannot be controlled in immunocompromised flies (24). Therefore, using this system, we sought to distinguish between pathology resulting from the immune response and pathology resulting from the microbe. We test whether the sexes are differentially impacted by these two sources of pathology using multiple metabolic and physiological measures as readouts. We show that females reduce the cost of immune activity via strict regulation of the immune deficiency (IMD) pathway and that this comes at the cost of bacterial clearance.

## Results

### Male and Female Flies Exhibit Differences in IMD Pathway Function after Infection.

**Fig. 1.**
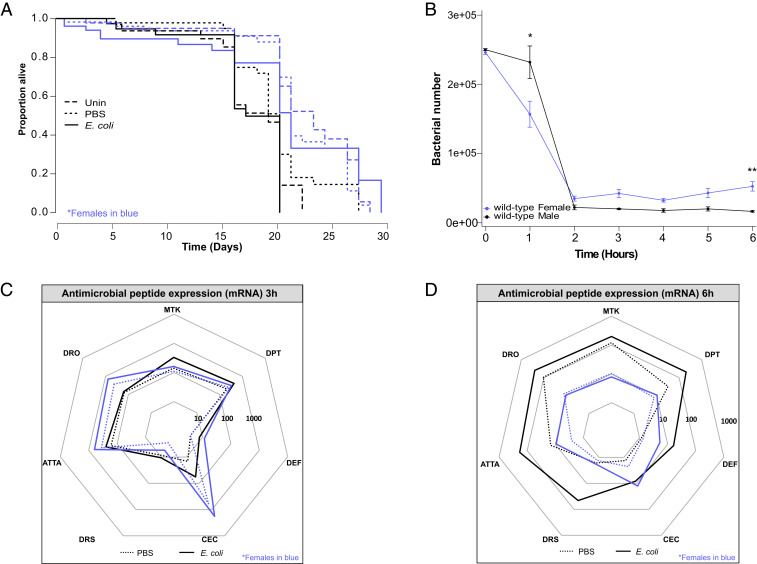
Sex-specific outcomes of *E. coli* infection. Representation in all plots: males, black; females, blue. (*A*) Survival of *E. coli*–infected wild-type flies. *E. coli*–infected flies are indicated by solid lines. Uninfected and PBS controls are indicated by long and short dashed lines, respectively. Flies had an average median survival across all treatments of 21.5 and 18.5 d for females and males, respectively (Coxph: degrees of freedom [df] = 7, *n* = 396, Wald test = 43.75, *P* = 2 × 10^−7^). There was no effect of treatment on survival in either sex. Survivals were performed at least twice, each repeat included 20 to 40 flies/treatment. (*B*) Bacterial quantification in wild-type flies. Females had more bacteria than males at 3 (Wilcoxon: W = 120, *P* = 0.019, *n* = 25), 4 (Student’s *t* test = 2.71, *P* = 0.013, *n* = 25), and 6 h (Wilcoxon: W = 148, *P* = 1.1 × 10^−4^, *n* = 25) postinjection. Markers indicate means, and bars represent SE. Statistical significance: *P < 0.05; ***P < 0.001. Quantifications were performed twice, each repeat included six to eight biological replicates consisting of one fly each. (*C* and *D*) AMP transcript levels 3 (*C*) and 6 h (*D*) postinfection in wild-type flies. Expression is shown relative to uninfected flies of the same sex. On average, infected males had AMP transcript levels 16× greater than females (*Mtk-*25x; *DptA*-19x; *Def*-3x; *CecA1*-0.65x; *Drs*-25x; *AttaA*-19x; *Dro*-23x). Solid lines represent infection with *E. coli*, while dotted lines are PBS injected. The area contained within the innermost heptagon represents induction levels falling between 1 and 10 times that of the uninfected controls. The middle and outer heptagons represent 100- and 1,000-fold induction, respectively. These data are also shown, represented differently, in *SI Appendix*, Fig. S1. AMP assays were performed two to four times, each repeat included three or four biological replicates/treatment consisting of three flies each.

To determine whether male and female flies exhibited a difference in their ability to defend against nonpathogenic gram-negative bacterial infection, we first measured survival and bacterial numbers after infection with *E. coli* of *w*^1118^ flies (henceforth referred to as “wild-type” because they have an intact immune response). Previous work has found that *D. melanogaster* infected with *E. coli* either eliminate the bacteria or maintain them at low levels at no obvious cost to the host (25, 26). As expected, we did not find a strong effect of infection with live or dead (heat killed) *E. coli* on the lifespan of wild-type flies (Fig. 1*A* and *SI Appendix*, Fig. S1*A* and Table S1). However, when we compared bacterial numbers between infected males and females, we found a clear trend toward greater numbers of surviving bacteria in females, which was significantly different at 3, 4, and 6 h following infection (Fig. 1*B*).

Defense against *E. coli* infection is expected to depend primarily on the activity of the IMD signaling pathway and its AMP target genes (27, 28). The fact that males and females exhibited differences in bacterial numbers led us to examine AMP mRNA expression 3 and 6 h after infection; these times were chosen because 3 h was not long after the bulk of bacterial killing had been achieved, while 6 h is the reported peak of *Diptericin* induction—a canonical read-out of *imd* activity—in wild-type animals (29). At 3 h after infection, male and female flies exhibited broadly similar levels of AMP transcripts (Fig. 1*C* and *SI Appendix*, Fig. S1*B*). However, by 6 h after infection, AMP expression was significantly reduced in female flies relative to males, despite females having higher bacterial numbers (Fig. 1*D* and *SI Appendix*, Fig. S1*C*). Importantly, AMP levels were notably greater in infected females 3 h following injection than they were at 6 h, while male levels were unchanged, possibly as a result of females being more responsive to bacterial load as a cue to shut down immune activity.

### Loss of *imd* Reveals Sex-Specific Tolerance to *E. coli* Infection.

**Fig. 2.**
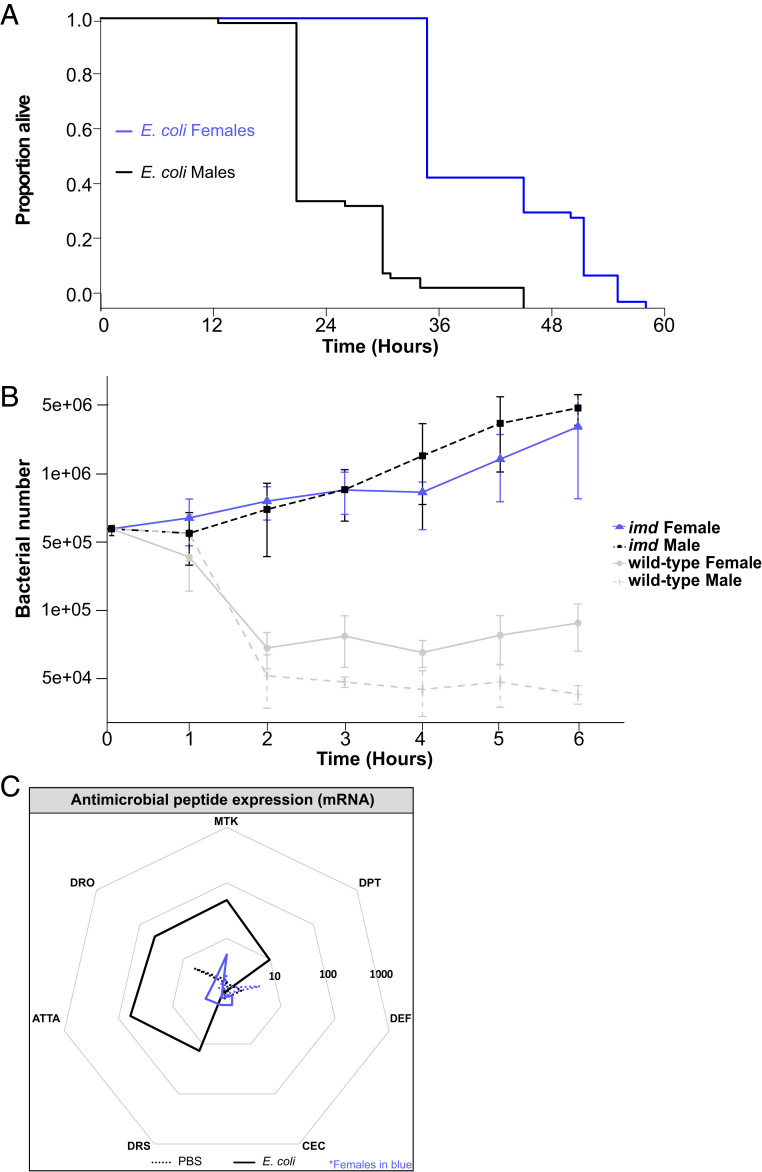
Sex-specific outcomes of *E. coli* infection in *imd* flies. Representation in all plots: males, black; females, blue. (*A*) Survival of *E. coli*–infected flies. Infected flies are indicated by solid lines. Median survival of *E. coli*–infected *imd* flies was 34 and 17 h for females and males, respectively (Coxph: df = 9, *n* = 255, Wald test = 126.2, *P* < 2 × 10^−16^). Survivals were performed at least twice, each repeat included 20 to 40 flies/treatment. Uninfected and PBS controls are here excluded for better visualization of the sex difference in survival. Survival of uninfected and wounded controls did not differ between the sexes during the assayed time (Log-Rank pairwise test: uninfected: *P* = 0.634; PBS: *P* = 0.198). Full survival including uninfected and PBS and controls shown in *SI Appendix*, Fig. S2. (*B*) Bacterial quantification in *imd* mutant flies. With the exception of 5 h postinfection when males had significantly more bacteria than females (Wilcoxon: W = 28.5, *P* = 4.4 × 10^−3^, *n* = 26), *imd* flies exhibited no difference in bacterial number between the sexes. Wild-type quantifications performed in tandem with *imd* flies are indicated in gray; note that this represents the same data shown in Fig. 1, and is repeated here to enable easy comparison. Markers indicate means, and bars represent SE. Statistical significance: **P < 0.01 Quantifications were performed twice, each repeat included six to eight biological replicates consisting of one fly each. (*C*) AMP transcript levels 6 h postinfection in *imd* mutant flies. Expression is shown relative to uninfected flies of the same genotype/sex. Solid lines represent infection with *E. coli*, while dotted lines are PBS injected. The area contained within the innermost heptagon represents induction levels falling between 1 and 10 times that of the uninfected controls. The middle and outer heptagons represent 100- and 1,000-fold induction, respectively. These data are also shown, represented differently, in *SI Appendix*, Fig. S2. AMP assays were performed two to four times, each repeat included three or four biological replicates/treatment consisting of three flies each.

The fact that we found the regulation of IMD signaling was different between the sexes led us to look more closely at the sex-specific consequences of the loss of *imd* function during *E. coli* infection. We infected *imd* mutants with *E. coli* and found that both sexes had significantly reduced survival when infected with *E. coli* compared to their phosphate-buffered saline (PBS)-injected and uninfected controls; infected *imd* males had a median survival only 60% that of *imd* females (Fig. 2*A* and *SI Appendix*, Fig. S2*A*). We then injected *imd* mutants with latex beads to inhibit their ability to phagocytose bacteria (30), resulting in flies with both phagocytosis and AMP activity inhibited; inhibiting the phagocytic response with latex beads did not affect survival in either sex (*SI Appendix*, Fig. S2*B*), further supporting the idea that AMP activity plays the primary role in this infection. When we examined bacterial loads in male and female *imd* mutants, we found that both sexes carried similar numbers of bacteria at all but one measured time, indicating that the difference in survival between male and female animals reflected different levels of infection tolerance (Fig. 2*B*). The fact that this differential tolerance effect was revealed only in *imd* mutants implied that it was a consequence of different components of non–IMD pathway immune activation and that the secondary immune response pathways revealed by *imd* mutation were more damaging to males, possibly because of quantitative differences in their activation between the sexes.

We tested this possibility by assaying AMP induction in *imd* mutants infected with *E. coli*. Females exhibited no response at all, while males exhibited a residual 10- to 100-fold induction of most AMPs (Fig. 2*C* and *SI Appendix*, Fig. S2). This level of expression was clearly insufficient for antimicrobial activity, as the sexes exhibited similar bacterial numbers but was potentially enough to cause pathology in *imd* mutant males.

### Infection with *E. coli* Leads to Depletion of Triglycerides.

Because resources are finite, individuals must manage investments in multiple biological processes. The ability to draw on metabolic reserves of triglyceride or glycogen allows animals to run temporary metabolic deficits in response to unexpected costs (e.g., immunity). We hypothesized that the sex differences we observed in immune activity and tolerance of infection in wild-type and *imd* mutant flies, respectively, might also be reflected in differences in the metabolic cost of infection. To test this, we assayed levels of free sugar (glucose and trehalose), stored carbohydrate (glycogen), stored triglyceride, and respiration in wild-type and *imd* flies. Previous studies in *D. melanogaster* found that lethal bacterial infections can lead to hyperglycemia, as well as a reduction in triglyceride and glycogen stores, but these metabolites had not been examined during acute infection with nonpathogens (31–33).

**Fig. 3.**
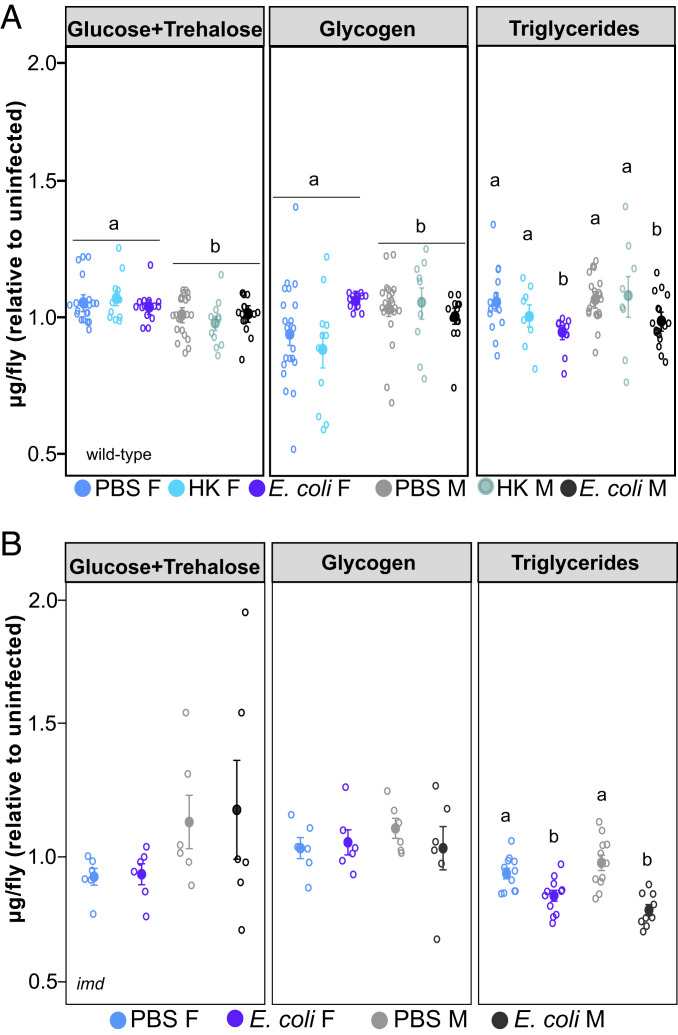
No sex difference in metabolic pathology of *E. coli* infection in wild-type and *imd* flies. Triglyceride and carbohydrate levels in live and heat-killed *E. coli*–infected flies. Data shown are quantities normalized to the mean of uninfected sex-matched controls. (*A*) In wild type, we employed an analysis of variance (AOV) there was an effect of sex on circulating sugar (AOV: df = 1, *n* = 67, F = 14.7, *P* = 2.7 × 10^−4^) and glycogen levels (AOV: df = 1, *n* = 59, F = 6.15, *P* = 0.016) with males having less circulating sugar but more glycogen stores than females. There was also an effect of infection status on triglyceride levels, such that *E. coli* infection led to triglyceride loss (AOV: df = 2, *n* = 74, F = 5.73, *P* = 4.8 × 10^−2^). There was no interaction between sex and infection on triglyceride loss. (*B*) *imd* flies showed no effect of sex nor infection status on circulating sugar and glycogen levels. There was no effect of sex on triglyceride levels, but there was an overall effect of both treatment (AOV: df = 1, *n* = 49, F = 44.971, *P* = 2.8 × 10^−8^) and the interaction between sex and treatment on triglycerides; *E. coli* infection led to triglyceride depletion in both sexes, relative to their PBS controls (AOV: df = 1, *n* = 49, F = 7.417, *P* = 9.2 × 10^−3^; male PBS-male [PBS-M] *E. coli*, *P* adjusted = 4.0 × 10^−7^; female PBS-female [PBS-F] *E. coli P* adjusted = 1.01 × 10^−2^). Bars indicate SE. Letters indicate statistical groupings. Full statistics including nonsignificant results can be found in *SI Appendix*, Table S2. All assays were performed two or three times, each repeat included four biological replicates/treatment consisting of three (carbohydrates) or eight (triglycerides) flies each.

We found that 6 h postinfection with *E. coli*, wild-type flies had significantly less stored triglyceride than their PBS controls; this effect was independent of sex (Fig. 3*A* and *SI Appendix*, Table S2). Importantly, infection with heat-killed *E. coli* did not deplete triglyceride, indicating that this effect is dependent on the presence of live bacteria and not merely on general immune activation. Wild-type males had significantly less circulating sugar but more glycogen than females, but neither of these was changed by infection. Respiration was unaffected by infection status in wild-type flies (*SI Appendix*, Fig. S3). *imd* mutants exhibited a somewhat different pattern to wild type, as there was no effect of infection on free sugar levels nor glycogen in either sex (Fig. 3*B*). As in wild-type flies, both male and female *imd* mutants exhibited significant reduction in triglyceride resulting from infection, and this effect was notably stronger in males (26 versus 13% less than PBS controls for males and females, respectively; Fig. 3*B* and *SI Appendix*, Table S2). This fit with our observation that *imd* mutant males exhibited a stronger (though clearly ineffective) immune response to *E. coli* infection than *imd* mutant females, as a possible cause for greater triglyceride depletion in males could be increased demands resulting from immune activity. Alternatively, males could be diverting resources into other, non–immune-related activities, such as foraging or reproduction (34, 35).

Because animals spend significant energy on reproduction, and reproductive effort is likely to restrict or trade-off with immunity (36), we assayed reproductive output during infection. We placed infected flies in tubes with flies of the opposite sex and “competitors” of the same sex but of a different genotype (*Dh44*[3xP3-DsRed]). We allowed flies to mate for 12 h and then discarded adults. Offspring resulting from matings with competitors were easily identifiable by their red-fluorescent eyes. Both wild-type and *imd* males were less likely to have a successful mating interaction than their female counterparts, but neither sex showed an effect of infection on mating success or the number of offspring produced (*SI Appendix*, Fig. S4). These findings demonstrate that despite observing metabolic shifts and sex-specific AMP induction and pathology (bacterial load), reproductive output is unaffected in the short term by *E. coli* infection.

### Sex-Specific Expression of IMD Pathway Regulators.

We have shown that male and female flies exhibit clear differences in the dynamics of the transcriptional response to *E. coli* infection, presumably due to distinct mechanisms of immune regulation and that in flies lacking the IMD pathway, male animals exhibit distinctly greater responses to infection in terms of gene expression and triglyceride depletion and die more rapidly than females. We wished to gain some mechanistic insight into these differences between the sexes, so we analyzed the expression of known negative regulators of IMD signaling in male and female flies. We expected that negative regulators responsible for the effects we observed on AMP expression should be more inducible in females.

**Fig. 4.**
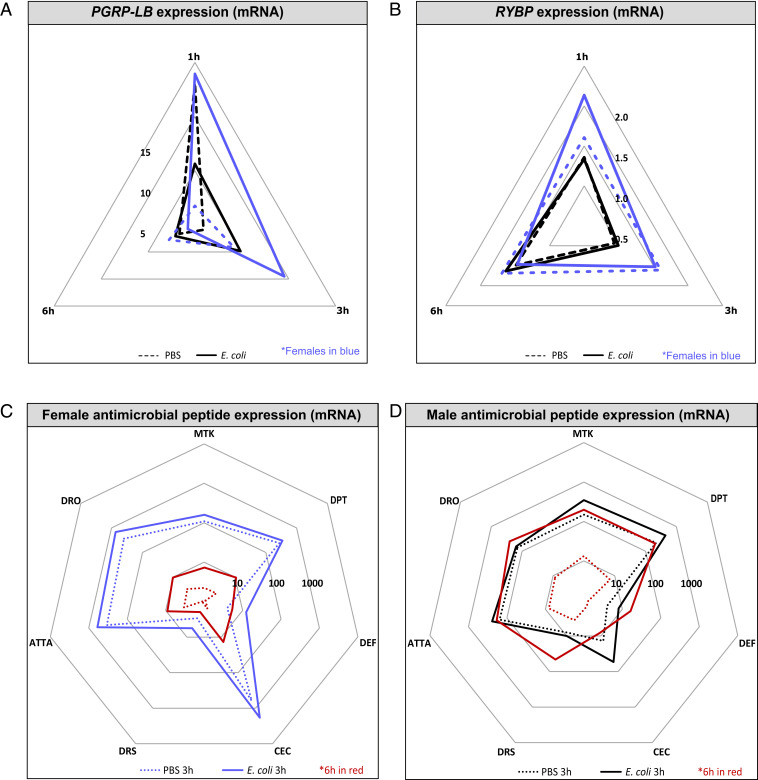
Sex-specific temporal regulation of *imd* during *E. coli* infection wild-type flies. Representation in all plots: males, black; females, blue. (*A* and *B*) Expression of *PGRP-LB* (*A*) and *RYBP* (*B*) 1, 3, and 6 h after infection in male and female flies. Plotted values are relative to the uninfected controls. Solid lines represent infection with *E. coli*, while dotted lines represent PBS injection. These data are also shown, represented differently, in *SI Appendix*, Fig. S5*B*. (*C* and *D*) AMP transcript levels in females (*C*) and males (*D*) 3 and 6 h after infection. Expression is shown relative to uninfected flies of the same genotype/sex. Solid lines represent infection with *E. coli*, while lines dotted are PBS injected. Data collected at 6 h are indicated in red. The area contained within the innermost heptagon represents induction levels falling between 1 and 10 times that of the uninfected controls (down-regulation was not observed in any of the tested genes). The middle and outer heptagons represent 100- and 1,000-fold induction, respectively. AMP assays were performed two to four times, each repeat included three or four biological replicates/treatment consisting of three flies each.

Several negative regulators of IMD pathway activity have been described (37–40). We assayed several of these regulators for increased infection inducibility in female flies relative to males (*SI Appendix*, Fig. S5*A*). Two negative regulators—*PGRP-LB* and *RYBP*—were expressed at higher levels specifically in *E. coli*–infected females 3 h postinfection (Fig. 4 *A* and *B*). A more-detailed analysis of the time course of expression of *PGRP-LB* and *RYBP* revealed that both were up-regulated as early as 1 h after infection in females, and both showed continuing strong expression 3 h after infection, especially in females (Fig. 4 *A* and *B* and *SI Appendix*, Fig. S5*B*). However, by 6 h after infection, *PGRP-LB* expression had returned to near normal in both males and females, while RYBP expression was now induced in males to the same high level seen from 1 h in females. This difference in the regulatory timing of the IMD pathway can be seen when we compare AMP expression at 3 and 6 h in each sex (Fig. 4 *C* and *D*).

PGRP-LB is an amidase that degrades the DAP-type peptidoglycan of gram-negative bacteria, dampening activation of the IMD pathway by degrading the activating ligand (40). In contrast, *RYBP* inhibits IMD pathway activity by promoting proteasomal degradation of the pathway’s NF-κB transcription factor, *Relish* (38). *PGRP-LB* reduces pathway activity by degrading free peptidoglycan—that is, it reduces pathway activity only when the immune response has been effective in killing bacteria; it was thus particularly interesting because its activation upon infection renders the IMD pathway responsive to its own success. Peptidoglycan-degrading activity also could regulate IMD-independent immune responses, which could explain the sex differences we observed in immune activity, metabolic impact, and infection pathology in *imd* mutants. We thus decided to analyze immune function in male and female *PGRP-LB* mutants.

### *PGRP-LB*^*Δ*^ Mutants Exhibit Reversed Sex Bias in Immunity, Improved Immune Function, and Altered Metabolic Response to Infection.

**Fig. 5.**
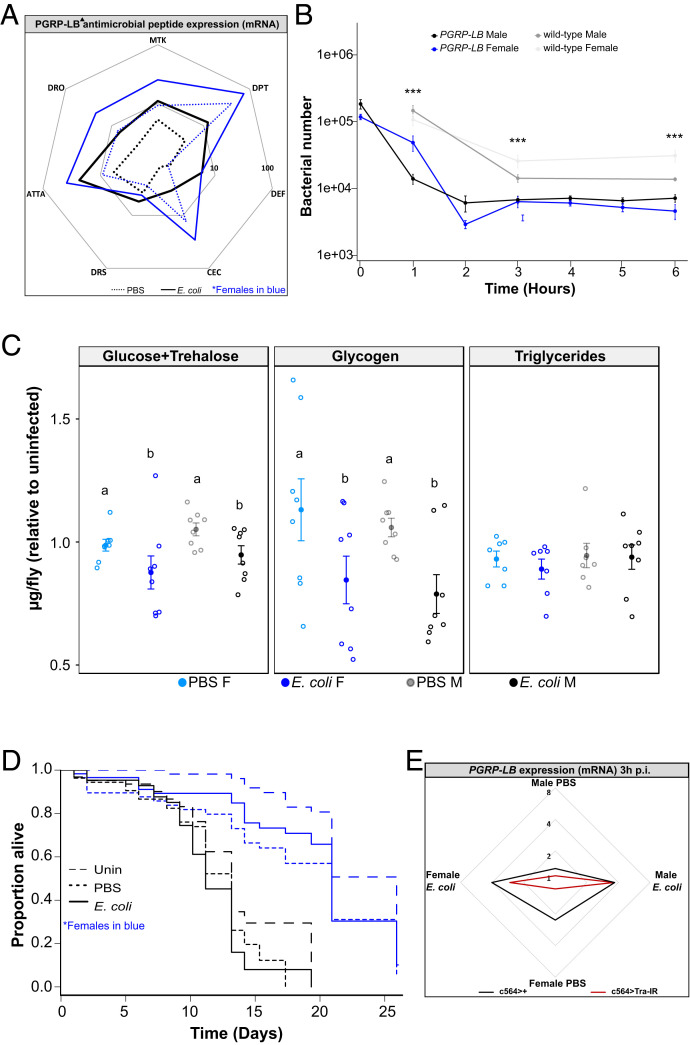
*PGRP-LB*^*Δ*^ males and females exhibit parallel metabolic shifts during infection. Representation in all plots: males, black; females, blue. (*A*) AMP expression is shown relative to uninfected flies of the same genotype/sex. Solid lines represent infection with *E. coli*, while dotted lines are PBS injected. The area contained within the innermost heptagon represents induction levels falling between 1 and 10 times that of the uninfected controls. The outer heptagon represents 100-fold induction. Assays were performed twice, each repeat included three to four biological replicates/treatment consisting of three flies each. These data are also shown, represented differently, in *SI Appendix*, Fig. S6*A*. (*B*) Bacterial load observed over the first 6 h of infection in *PGRP-LB*^*Δ*^ flies did not differ between the sexes. Wild-type quantifications were performed in tandem with *PGRP-LB*^*Δ*^ flies at select time points (1 , 3, and 6 h) and are indicated in gray. Wild-type flies had significantly more bacteria than *PGRP-LB*^*Δ*^ at all points measured (1 h: Kruskal–Wallis = 24.8, *P* = 1.7 × 10^−5^, *n* = 79; 3 h Kruskal–Wallis = 18.4, *P* = 3.7 × 10^−4^, *n* = 70; 6 h Kruskal–Wallis = 30.7, *P* = 9.9 × 10^−7^, *n* = 55). Markers indicate means, and bars represent SE. Statistical significance: *P < 0.05; **P < 0.01; ***P < 0.001. Quantifications were performed two to four times, each repeat included five to eight biological replicates consisting of one fly each. (*C*) Infection had a significant effect on circulating sugar such that the amount of circulating sugar in *E. coli*–infected animals was lower than in PBS controls (AOV: df = 1, *n* = 32, F = 6.44, *P* = 1.7 × 10^−2^), whereas sex had no effect on circulating sugars, nor was there a significant interaction between the two. Similarly, *E. coli* infection led to marked reduction in stored glycogen (AOV: df = 1, *n* = 32, F = 9.41, *P* = 4.8 × 10^−3^), with no effect of sex, nor a significant interaction between sex and treatment. Neither infection status nor sex effected triglyceride levels. Large, filled markers indicate means, while smaller circles represent individual data points. Letters indicate statistical groupings. Bars indicate SE. All assays were performed twice, each repeat included four biological replicates/treatment consisting of three (carbohydrates) or eight (triglycerides) flies each. Full statistics including nonsignificant results can be found in *SI Appendix*, Table S3. (*D*) Survival of flies infected with *E. coli* indicated by solid lines. Uninfected and PBS controls are indicated by long and short dashed lines, respectively. *E. coli*–infected females had a median survival 58% greater than that of males (Female = 20.9 d, Male = 13.2 d; Coxph: df = 5, *n* = 484, Wald test = 119, *P* = 2.0 × 10^−16^). Survivals were repeated thrice, each repeat included one or two biological replicates/treatment consisting of 20 flies each (note that data after day 21 represent two repeats). Full survival including uninfected wild-type controls is shown in *SI Appendix*, Fig. S7*B*. (*E*) *PGRP-LB* expression 3 h postinfection (p.i.) with *E. coli* in flies with *tra* knocked down in the fat body. Data are shown relative to uninfected flies of the same genotype/sex. Solid lines represent infection with *E. coli*, while dotted are PBS injected. Red and black tracings show *tra* knock down in the fat body and driver control, respectively. The area contained within the innermost heptagon represents induction levels falling between one and two times that of the uninfected controls. The outer heptagon represents eightfold induction. Assays were performed twice, each repeat included three to four biological replicates/treatment consisting of three flies each. These data are also shown, represented differently, in *SI Appendix*, Fig. S7*C*.

To test whether *PGRP-LB* activity was responsible for the sex difference in immune function, we infected male and female *PGRP-LB* null mutants with *E. coli* and measured AMP expression, bacterial numbers, and survival of the host. In the absence of *PGRP-LB*, the male-biased AMP expression observed 6 h following infection with *E. coli* in wild-type flies was abolished (Fig. 5*A* and *SI Appendix*, Fig. S6*A*). *PGRP-LB*^*Δ*^ mutants had fewer bacteria than wild type at all time points assayed (1, 3, and 6 h; Fig. 5*B*). As in wild-type flies, *PGRP-LB*^*Δ*^ mutants of both sexes drastically reduced bacterial load within the first 2 h postinfection, at which time bacterial numbers effectively plateaued. However, in contrast to what we saw in wild-type flies, *PGRP-LB*^*Δ*^ females did not carry higher bacterial loads than males at any point throughout the 6-h period assayed (Fig. 5*B*), confirming our supposition that wild-type females down-regulate AMP activity at a cost of resistance, and indicating that sex-specific *PGRP-LB* induction has important functional consequences for the realized immune response.

We next aimed to identify the effects of *PGRP-LB* on the physiological consequences of immune activation—in particular, to explore the extent to which the metabolic consequences of acute infection are driven by host- or pathogen-derived activities. We predicted that if triglyceride loss observed in both sexes during *E. coli* infection in wild-type flies is driven entirely by pathogen-derived costs that the reduced bacterial load observed in infected *PGRP-LB*^*Δ*^ flies might be sufficient to abrogate triglyceride loss; conversely, if triglyceride loss were driven by IMD pathway activity, the prolonged IMD pathway activation observed in *PGRP-LB* mutants should result in greater loss of triglyceride than in wild-type animals. We found that in both male and female *PGRP-LB*^*Δ*^ flies, triglyceride levels were unaffected by *E. coli* infection, confirming that something other than IMD pathway activity causes triglyceride depletion in this infection. Infected *PGRP-LB*^*Δ*^ flies of both sexes had lower levels of circulating sugars and glycogen (Fig. 5*C* and *SI Appendix*, Table S3). This effect of infection on circulating and mobile energy observed in *PGRP-LB*^*Δ*^ flies may be indicative of the energy requirement of an unabated immune response.

The effect on overall lifespan was more complex: similar to what we observed in wild-type flies, independent of infection status, *PGRP-LB*^*Δ*^ females lived longer than males (Fig. 5*D*). Wounding had a significant impact on survival in females, with both PBS- and *E. coli*–injected animals having reduced survival (though the two treatments did not differ from each other). Because PGRP-LB should have little effect in the absence of peptidoglycan, the effect of sterile wounding in females was somewhat confusing; one possibility is that the previously documented effect of PGRP-LB on interaction with microbiota-derived peptidoglycan may have specific importance in the regulation of immune responses following sterile injury (41).

### Fat Body *transformer* and IMD Pathway Signaling Promote *PGRP-LB* Expression.

We wished to determine the roles of sex-specific regulatory factors and immune pathway activation in driving the female-specific *PGRP-LB* induction seen after *E. coli* infection. The gene *transformer* (*tra*) is part of the regulatory pathway responsible for female sex determination in *D. melanogaster*. Functional Tra protein is produced only in females and is necessary for most female-specific gene expression in somatic tissues and consequently for several sex-specific traits related to growth, metabolism, and aging pathologies (42–45). Since *E. coli* peptidoglycan activates the IMD pathway, leading to the synthesis and secretion of AMPs by the fat body (24, 27, 46, 47), and *PGRP-LB* degrades peptidoglycan to prevent IMD pathway activation, we decided to knockdown *tra* in the fat body to test its requirement in sex-specific regulation of *PGRP-LB*. At 3 h after injection, PBS- and *E. coli*–infected females with *tra* knocked down (c564 > tra-IR) had reduced *PGRP-LB* expression relative to their genetic controls and males of the same treatment (Fig. 5*E* and *SI Appendix*, Table S5). As expected, *tra* knockdown in males had no effect on *PGRP-LB* expression. The IMD pathway is required for most *E. coli*–induced gene expression; we tested *PGRP-LB* expression in *imd* mutant flies and found that *E. coli* infection did not induce *PGRP-LB* expression in these animals (*SI Appendix*, Fig. S6*B*). These findings demonstrate that *PGRP-LB* expression is driven via combined inputs from *tra* and the IMD pathway, resulting in female-specific transcriptional induction of this regulator after infection.

## Discussion

Differences between males and females in immune activity and infection outcomes are pervasive throughout the animal kingdom. Here, we have explored the differences between male and female *Drosophila* in their response to a nonpathogenic gram-negative bacterial infection. Though both males and females could control this infection at the cost of only transient metabolic depletion, our analysis revealed that females maintained much-stricter control of their own immune response; this was achieved by female-specific transcriptional induction of a peptidoglycan amidase that degrades peptidoglycan fragments liberated from bacteria after they are killed, effectively enabling the female immune response to monitor its own effectiveness and to shut down when no longer needed. Elimination of this mechanism improved bacterial killing by the female immune response. Thus, indirect costs associated with infection (i.e., immune activity) rather than pathogen-derived effects drove these sex-specific immune outcomes. This is not the first demonstration of a difference in infection outcomes between the sexes originating from differential regulation of innate immune sensing; in mice, muting the inhibitory receptor CD200 resulted in greater immune activity and viral clearance, but this effect was more pronounced in female mice (48). However, this is a case in which differential immune regulation between the sexes results from differential degradation of microbial immune elicitors.

Stricter regulation of the IMD pathway by females suggests that immune activity may come at a greater burden to them. Uninfected wild-type females had a median survival 9.6% greater than females injected with PBS, heat-killed *E. coli*, and live *E. coli* (*SI Appendix*, Table S1). In contrast, only injection with live *E. coli* affected male survival (down 11.7% from uninfected). Because heat-killed *E. coli* are able to activate the immune response without causing mortality (shown here and in ref. 49), these findings indicate that immune activation comes at a greater cost to females. Together, these data support the idea that the IMD response is costly and that its activity poses a greater burden to females, leading to sex-specific differences in indirect—rather than pathogen-derived—pathology. An alternative idea is that the energy demand of *E. coli* infection in *PGRP-LB*^*Δ*^ flies, as indicated through the decrease in both circulating and stored carbohydrate, was pathogen derived rather than immune. Bacteria have been shown to utilize host resources during infection (15, 50, 51) and while this would be surprising in this infection as bacterial numbers were declining (and were also lower than in wild-type infection, in which carbohydrate loss was absent), it remains a possibility. Indeed, the depletion of circulating sugars and glycogen in *PGRP-LB*^*Δ*^ flies supports a model of pathogen-derived glycogenolysis (51).

Elimination of *PGRP-LB* resulted in increased expression of *diptericin* (an indicator of IMD pathway activity) and thus, unsurprisingly, *PGRP-LB*^*Δ*^ flies had fewer bacteria than wild-type over the first 6 h postinfection (Fig. 5*B* and *SI Appendix*, Fig. S7*A*). The absence of triglyceride loss in these animals, associated with increased immune responses and reduced microbial loads, suggests that in this infection, triglyceride is lost because of direct pathogen effects. We have recently shown that when flies infected with the gram-negative pathogen *Francisella novicida* were treated with antibiotics to keep bacterial numbers low, they did not exhibit infection-driven metabolic shifts (including triglyceride loss). In contrast, when bacterial numbers increased (still in the presence of antibiotic treatment), metabolic shifts during infection were again observed, suggesting that these changes were associated with bacterial load rather than being a direct effect of the antibiotics on metabolism (33).

The immune response, as we normally envision it, includes responses to infection that protect the host by killing pathogens or restricting their growth (resistance). In contrast, tolerance is defined as the ability to maintain health during infection. Experimentally, a more-tolerant host is one that remains healthy longer at a given pathogen load (52, 53). Recent years have seen increasing interest in tolerance, driven in part by the idea of improving tolerance as a therapeutic approach to infection. However, despite the large body of theory surrounding tolerance, the ability to detect tolerant phenotypes (54), and the identification of tolerance-associated genes (31, 52, 55), we still know very little about the fundamental mechanisms of tolerance. It has previously been shown that *PGRP-LB* contributes to infection tolerance (40); we show that this activity is in fact sexually dimorphic. Importantly, through our finding that the masculinization of the female fat body led to a reduction in *PGRP-LB* expression (Fig. 5*E*), this work also demonstrates that the sexually dimorphic *PGRP-LB* activity is mediated by sex-determinant pathways. Furthermore, we show that phenomenological differences in tolerance between the sexes can be used to identify fundamental mechanisms of infection tolerance and that the sex-specific regulation of inhibitors of immune signaling can underlie strong, complex differences in immune dynamics between the sexes.

## Materials and Methods

### *Drosophila* Genetics and Culture.

*w*^1118^ flies and *w*^1118^; *imd*^10191^ were used as wild-type and IMD pathway mutants, respectively. The *imd*^10191^ line carries a 26-nucleotide deletion that frameshifts the IMD protein at amino acid 179, which is the beginning of the death domain (56). *PGRP-LB*^∆^ mutant lines used were obtained from the Bloomington Stock Center and have been previously described (57). Both *imd*^10191^ and *PGRP-LB*^∆^ were placed on our *w*^1118^ genetic background using isogenic balancer chromosome lines. For *tra* knockdown experiments, we used *w*^1118^; *c564-Gal4* (fat body driver) and *w*^1118^; *UAS-tra2-RNAi* from Bloomington *Drosophila* Stock Center and the Vienna *Drosophila* Resource Center, respectively. Flies were maintained on a sugar-yeast diet (10% wt/vol autolyzed brewer’s yeast, 8% fructose, 2% polenta, and 0.8% agar, supplemented with 0.075% wt/vol nipagin and 0.75% vol/vol propionic acid) at 25 °C.

### *Drosophila* Infection.

For all experiments, flies were collected within 24 h following eclosion and kept in same-sex vials for 5 to 7 d in groups of 20. Thus, all experiments were conducted on flies between 5 and 8 d old. Injections were carried out using a pulled-glass capillary needle and a Picospritzer injector system (Parker). Following injection, flies were kept at 29 °C. Bacteria were grown from single colonies overnight at 37 °C shaking. Each fly was injected with 50 nL of *E. coli* suspended in PBS (optical density at 600 nm [OD_600_] = 1.0 ∼100,000 bacteria). Following resuspension in PBS, a subset of bacteria designated for the “heat-killed” treatment was incubated for 1 h at 65 °C. Sterile PBS was used as a wounding control. A subset of *imd* flies were preinjected with 0.2-µm latex beads, FluoSpheres, Carboxylate-Modified Microspheres (Invitrogen) to inhibit phagocytosis as previously described (30, 56). Briefly, beads were washed 3× in sterile PBS and resuspended in PBS at one-fourth of the original volume of the bead stock. Flies were injected with 50 nL bead-PBS solution or PBS alone, left for 16 h, and then injected with PBS or *E. coli*.

### Survival Assays.

Survival experiments were performed at 29 °C with 15 to 20 flies/vial. Survival was monitored daily, and flies were tipped into fresh vials every 4 d.

### Bacterial Quantification.

For each sample, one fly was homogenized in 100 µL sterile ddH_2_O. Homogenates were serially diluted and plated onto Luria-Bertani (LB) agar plates where they incubated for 16 to 18 h. Following incubation, the number of individual bacterial colonies observed on each plate was quantified and back calculated to determine the number of colony-forming units (CFU) present in each fly. Individual fly quantifications are presented in *SI Appendix*, Fig. S8.

### Gene Expression—qRT-PCR.

For each sample, three flies were homogenized in 100 µL single-step RNA isolation reagent TRI Reagent (Sigma), followed by a chloroform extraction and precipitation in isopropanol. The resultant pellet was then washed with 70% ethanol. Pellets were resuspended and subject to DNase treatment. Revertaid M-MuLV reverse transcriptase and random hexamers (Thermo Fisher Scientific) were used to carry out complementary DNA (cDNA) synthesis. A volume of 5 µL from each cDNA sample was put into a “neat” standards tube; this tube was later used to generate standards which were used to generate a standard curve for each gene. Each cDNA sample was diluted and this diluted sample used for analysis.

We used Sensimix with SYBR Green no-ROX (Bioline) or qPCRBIO SyGreen Mix Separate-ROX (PCR Biosystems) for qRT-PCR. Reactions were run on a Corbett Rotor-Gene 6000 with cycling conditions as follows: Hold 95 °C for 10 min, then 45 cycles of 95 °C for 15 s, 59 °C for 30 s, and 72 °C for 30 s, followed by a melting curve. Primers used are listed in Table 1. Gene expression was calculated based on the standard curve generated during each run, normalized to the value of our housekeeping gene, *Rpl4*. Samples from PBS and infected treatments were then divided by the mean value of their uninfected controls to generate expression values relative to uninfected flies.

**Table 1.** Primer sequences used for qRT-PCR

Gene	Forward	Reverse
*AttA*	5′- CAC​AAT​GTG​GTG​GGT​CAG​G -3′	5′- GGCACCATGACCAGCATT -3′
*CecA1*	5′- TCT​TCG​TTT​TCG​TCG​CTC​TC -3′	5′- CTT​GTT​GAG​CGA​TTC​CCA​GT -3′
*Def*	5′- TTC​TCG​TGG​CTA​TCG​CTT​TT -3′	5′- GGA​GAG​TAG​GTC​GCA​TGT​GG -3′
*DptA*	5′- ACC​GCA​GTA​CCC​ACT​CAA​TC -3′	5′- CCC​AAG​TGC​TGT​CCA​TAT​CC -3′
*Dro*	5′- CCA​TCG​AGG​ATC​ACC​TGA​CT -3′	5′- CTTTAGGCGGGCAGAATG -3′
*Drs*	5′- GTA​CTT​GTT​CGC​CCT​CTT​CG -3′	5′- CTT​GCA​CAC​ACG​ACG​ACA​G -3′
*Mtk*	5′- TCT​TGG​AGC​GAT​TTT​TCT​GG -3′	5′- TCT​GCC​AGC​ACT​GAT​GTA​GC -3′
*Rpl4*	5′- TCC​ACC​TTG​AAG​AAG​GGC​TA -3′	5′- TTG​CGG​ATC​TCC​TCA​GAC​TT -3′
*PGRP-LB*	5′- TGA​TCG​GAG​ATT​GGA​GAA​CC -3′	5′- AAG​GCG​ATC​AGG​TTC​TTG​G -3′
*RYBP*	5′- GCGAAGGTGATCGAGGAG -3′	5′- GAG​TTC​AGG​CGT​GGC​TTT​C -3′

All gene expression experiments were performed at least twice, with three or more biological replicates per experiment.

### Measurement of Triglycerides.

Triglycerides were measured using thin layer chromatography (TLC) assays as described elsewhere (58). Briefly, each sample consisted of 10 flies; flies were placed in microcentrifuge tubes and stored at −80 °C until the time of analysis. To perform the TLC assay, samples were removed from the −80 °C freezer and spun down (3 min at 13,000 rpm at 4 °C) in 100 µL 3:1 (vol/vol) mix of chloroform and methanol. Flies were then homogenized and subject to a further “quick spin.” Standards were generated using lard dissolved in the same chloroform: methanol solution. We loaded 2 µL each standard and 20 µL each sample onto a silica gel glass plate (Millipore). Plates were then placed into a chamber preloaded with solvent (a 4:1 [vol/vol] mix of hexane and ethyl ether) and left to run until the solvent reached a point 1 cm short of the edge of the plate. Plates were then removed from the chamber, allowed to dry, and stained with cerium ammonium molybdate (CAM) solution (58). Plates were baked at 80 °C for 15 to 25 min and imaged using a scanner. Triglyceride was quantified in Image J using the Gel Analysis tool.

### Measurement of Carbohydrates (Glucose + Trehalose and Glycogen).

Each sample consisted of three flies that were homogenized in 75 μL Tris-EDTA buffer (TE) + 0.1% Triton X-100 (Sigma Aldrich). Samples were incubated for 20 min at 75 °C and stored at −80 °C. Prior to the assay, samples were incubated for 5 min at 65 °C. Following incubation, 10 μL from each sample was loaded into 4 wells of a 96-well plate. Each well was designated to serve as a measurement for either: control (10 μL sample + 190 μL H_2_0), glucose (10 μL sample + 190 μL glucose reagent [Sentinel Diagnostics]), trehalose (10 μL sample + 190 μL glucose reagent + trehalase [Sigma Aldrich]), or glycogen (10 μL sample + 190 μL glucose reagent + amyloglucosidase [Sigma Aldrich]). A standard curve was generated by serially diluting a glucose sample of known concentration and adding 190 μL glucose reagent to 10 μL each standard. Standards were always run at the same time and in the same plate as samples. Plates were incubated for 1.5 to 3 h at 37 °C, following which the absorbance for each well at 492 nm was determined using a plate reader.

### Respirometry.

Respiration in flies was measured using a stop-flow gas-exchange system (Q-Box RP1LP Low Range Respirometer, Qubit Systems). Eight flies from each treatment were put into an airtight glass tube and supplied with our standard fly food via a modified pipette tip. Each tube was provided with CO_2_-free air, while the “spent” air was concurrently flushed through the system and analyzed for its CO_2_ and O_2_ content. In this way, evolved CO_2_ and consumed O_2_ were measured for each tube every ∼44 min (the time required to go through each of the seven vials in sequence). For most replicates of the respirometry assay, there were two uninfected, two PBS, and three infected vials.

### Reproductive Assay.

Flies were collected within 7 h of eclosion to ensure virginity. To assess fitness, immediately following injection with either PBS or *E. coli*, flies were placed into vials with uninfected competitors of the same sex and potential mates of the opposite sex. Competitor flies expressed DsRed marker eyes; this marker allowed for easy identification of offspring resulting from focal flies—any DsRed-eyed offspring were the progeny of competitor flies. Flies were allowed to mate for 12 h, as this interval exceeds the time required for flies to significantly reduce the number of—and by some reports, clear—*E. coli*, thus allowing us to observe fitness throughout the infection. In one block, *E. coli* reproductive assays were left for 24 h; we have included these data, as number of offspring produced did not differ from the shorter assay, possibly because females do not lay many eggs overnight. After the mating period, flies were discarded, and vials were left for 14 d to allow resultant offspring time to develop and eclose.

### Statistical Analysis.

Data were analyzed in R Studio with R version 3.5.1 (59). Survival data were initially analyzed using Cox proportional hazards models; we then used Log-Rank tests for pairwise comparisons. We ran a generalized linear model (GLM) of reproductive success by sex and infection treatment; then, using only those matings resulting in offspring, we performed a GLM on number of offspring produced by sex and infection treatment. Detailed fitness and survival data have been deposited (60). For all other assays, we first tested for normality of data which dictated whether a factorial ANOVA model, Student’s *t* test, Kruskal–Wallis ANOVA, or Mann–Whitney U test was used to calculate differences between treatments with sex and infection status as factors. Initial models included experimental replicate as a factor, which was removed once we failed to observe an effect. When appropriate, we performed post hoc Tukey or Dunn analyses to identify specific differences between treatments.

## Supplementary Material

Supplementary File

## Data Availability

All data are provided in the paper and supplements; detailed fitness and survival data have been deposited at Research Data Repository (DOI: 10.14469/hpc/8546).
