# Supplementary material for: Disparate regulation of IMD signaling drives sex differences in infection pathology in Drosophila melanogaster
Source: Proc Natl Acad Sci U S A. 2021 Aug 2;118(32):e2026554118. doi: 10.1073/pnas.2026554118 (PMC8364183; doi:10.1073/pnas.2026554118)
Supplement: Supplementary File [file pnas.2026554118.sapp.pdf]

SUPPORTING INFORMATION for Vincent & Dionne, “Disparate regulation of IMD signaling drives sex differences in infection pathology in *Drosophila melanogaster*”,

This document contains eight supplementary figures (Fig S1-S8) and five supplementary tables (Tab S1-S5).

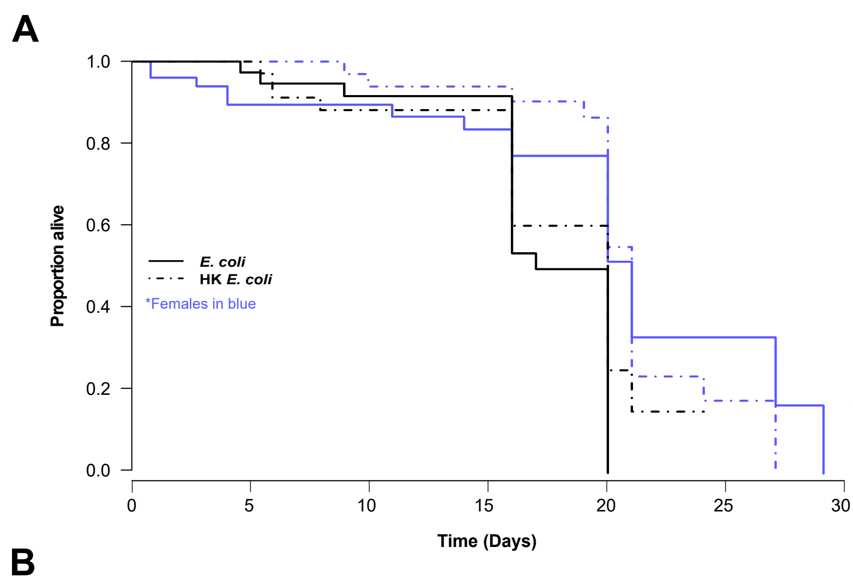

Figure S1. (A) Survival of wild-type flies infected with live and heat-killed (HK) *E. coli*. Males and females are represented by black and blue tracings, respectively. HK bacteria were incubated for 1h at 65 C. Live *E. coli* data are replotted from figure 1a. Antimicrobial peptide expression **(B)** 3h and **(C)** 6h following *E. coli* injection. All genes were standardized to the housekeeping gene *ribosomal protein 1*. Data are presented in arbitrary units. Markers represent individual data points. Bars indicate SE. All assays were performed twice, each repeat included 3-4 biological replicates/treatment consisting of 3 flies each.

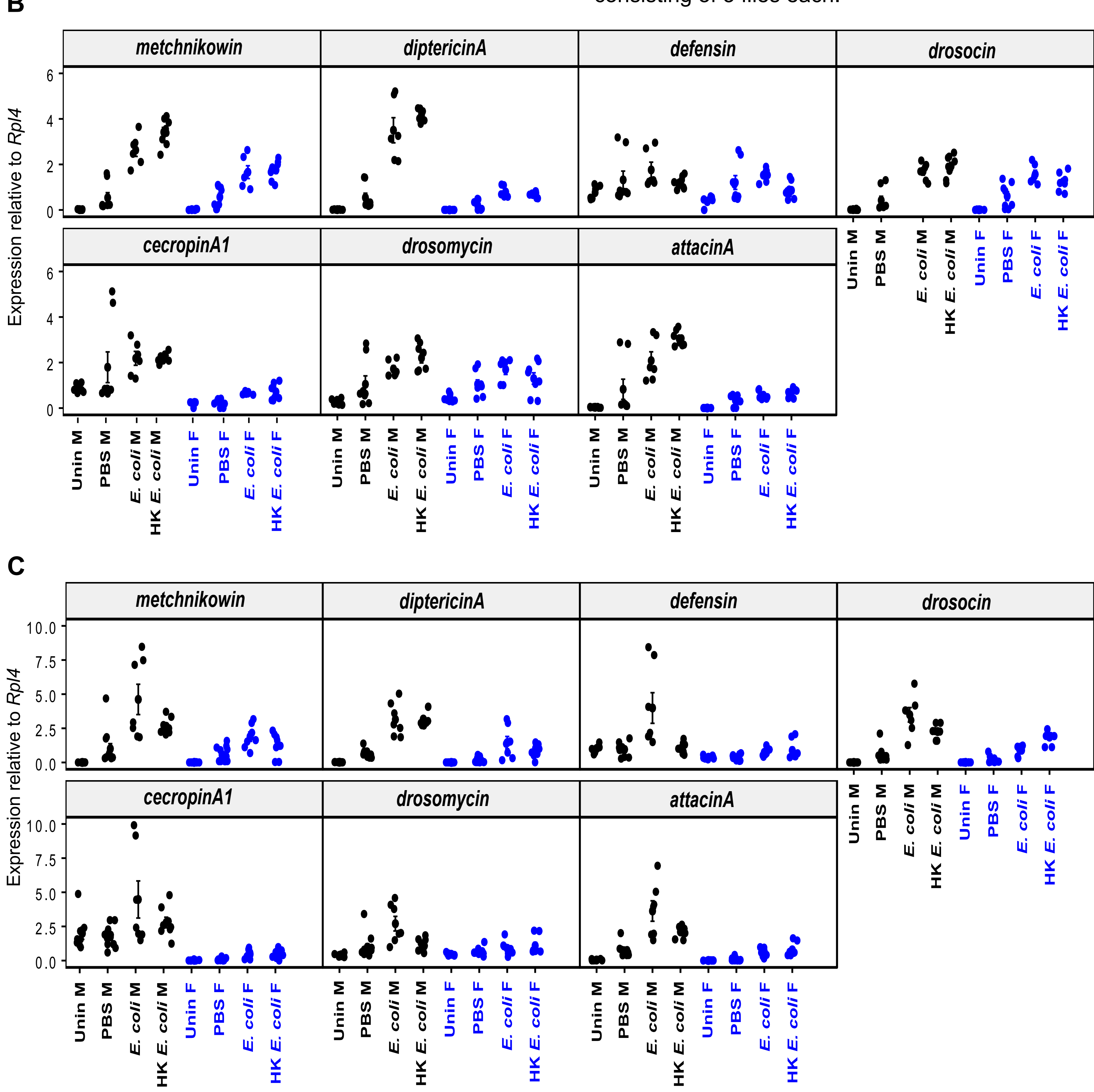

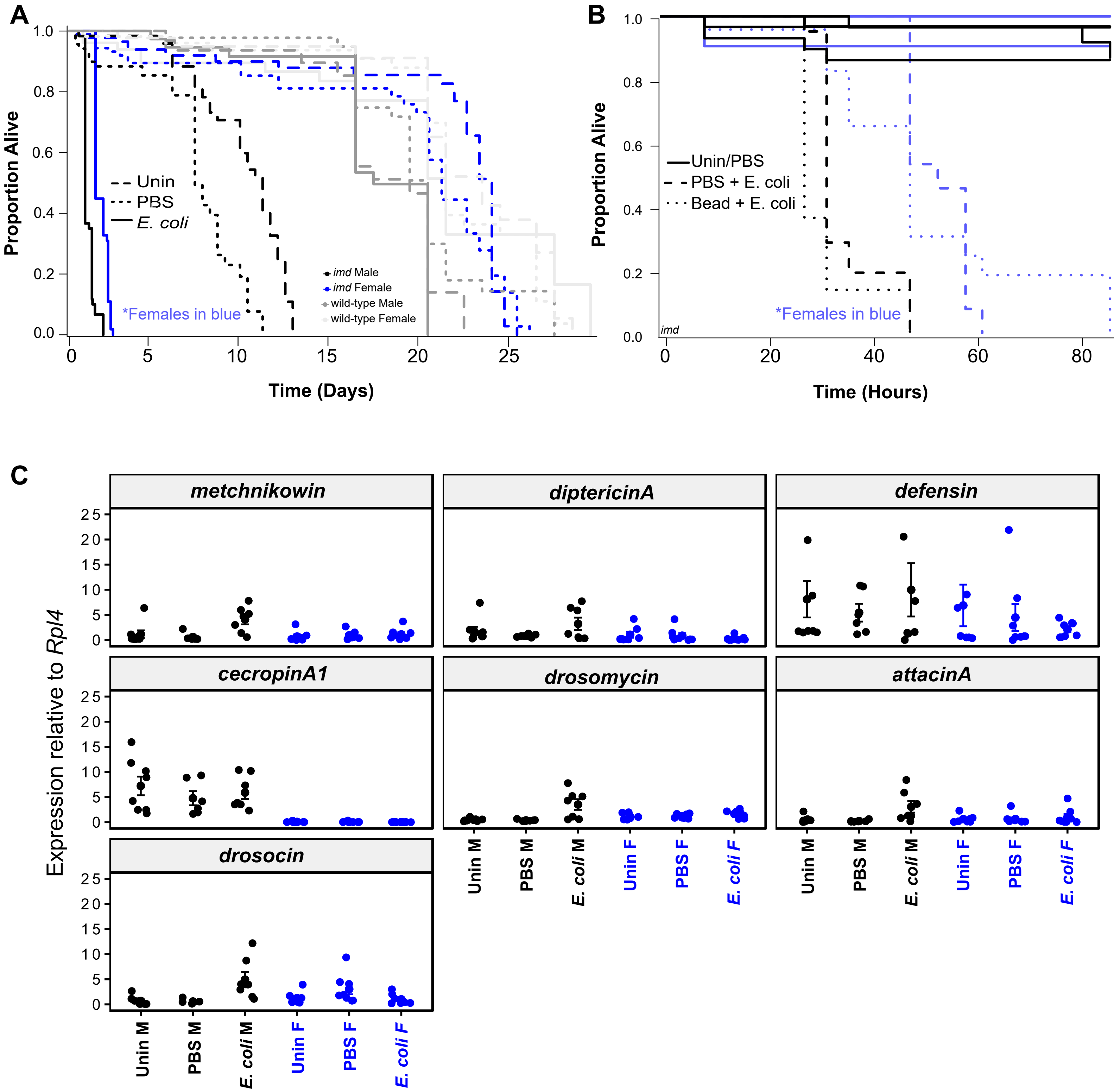

Figure S2. (A) Complete survival of *E. coli* infected flies. *Escherichia coli* infected flies are indicated by solid lines. Uninfected and PBS controls are indicated by long and short dashed lines, respectively. Both PBS and infection reduced male survival whilst female survival was only affected by infection. Wild-type males and females are represented by dark and light grey tracings, respectively. Survivals were performed at least twice, each repeat included 20-40 flies/treatment. **(B)** Survival of *imd* mutants pre-injected with either beads or PBS prior to *E. coli*. In boxplot, median value is indicated by horizontal bars, top and bottom of boxes represent upper and lower quartiles (respectively). Whiskers indicate maximum and minimum values. **(C)** Antimicrobial peptide expression 6h following *E. coli* injection in imd mutant flies. All genes were standardized to the housekeeping gene *ribosomal protein 1*. Data are presented in arbitrary units. Markers represent individual data points. Bars indicate SE. All assays were performed twice, each repeat included 3-4 biological replicates/treatment consisting of 3 flies each.

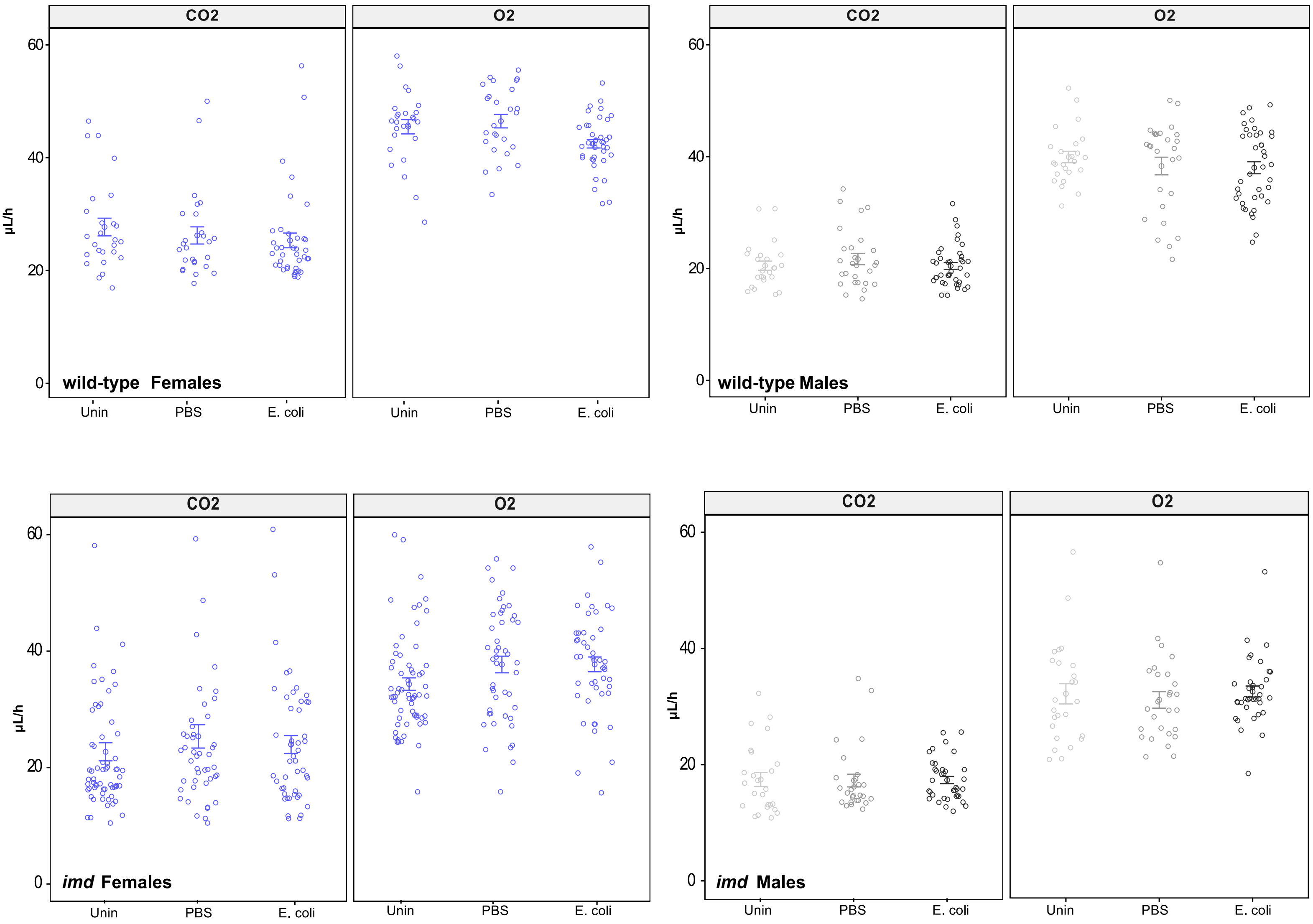

Figure S3. Respiration of infected males and females. Respiration was measured for six hours following infection. Markers represent one vial consisting of 8 flies. Bars indicate SE. All assays were repeated 3 or 4 times with 2 or 3 samples/treatment.

A

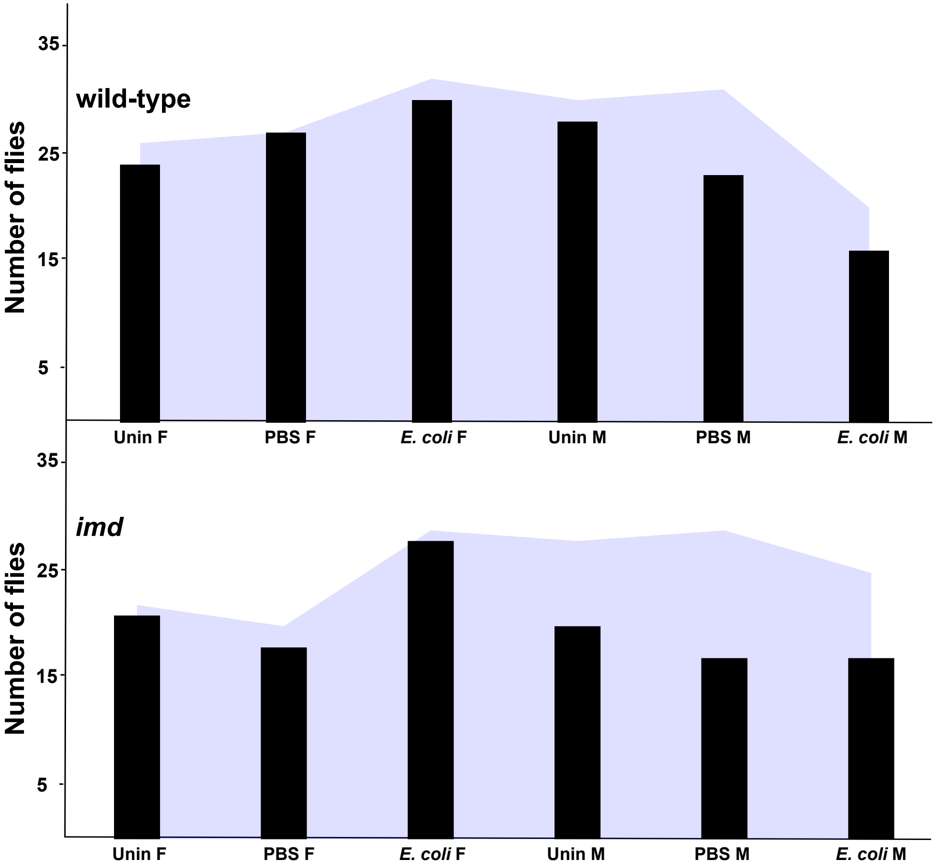

B

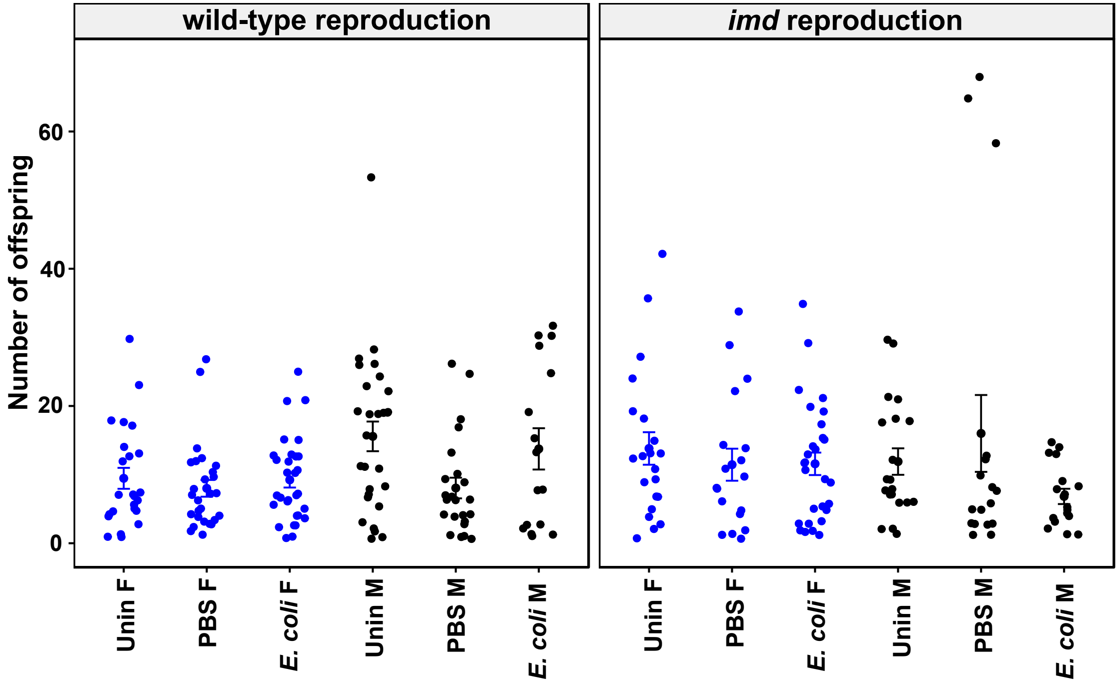

Coefficients (*w*^{1118})

Variable	Estimate	Std. Error	z Value	Pr(>|z|)
(Intercept)	2.823227	0.597194	4.727	2.27E-06
Sex Male	-1.499923	0.598868	-2.505	0.0123
Treatment PBS	0.004957	0.59761	0.008	0.9934
Treatment Unin	0.766838	0.695581	1.102	0.2703

Coefficients (*imd*^{10191})

Variable	Estimate	Std. Error	z Value	Pr(>|z|)
(Intercept)	2.9754	0.5969	4.985	6.19E-07
Sex Male	-2.1456	0.5679	-3.778	1.58E-04
Treatment PBS	-0.5373	0.5175	-1.038	0.299222
Treatment Unin	0.084	0.5497	0.153	0.878556

Coefficients (*w*^{1118})

Variable	Estimate	Std. Error	t Value	Pr(>|t|)
(Intercept)	9.233	1.552	5.948	2.02E-08
Treatment PBS	-1.233	2.256	-0.547	0.5854
Treatment Unin	0.225	2.329	0.097	0.9232
Sex Male	4.517	2.632	1.716	0.0884
Sex Male*Treatment PBS	-4.473	3.571	-1.253	0.2123
Sex Male*Treatment Unin	1.596	3.539	0.451	0.6526

Coefficients (*imd*^{10191})

Variable	Estimate	Std. Error	t Value	Pr(>|t|)
(Intercept)	11.571	2.252	5.139	1.14E-06
Treatment PBS	-0.127	3.6	-0.035	0.9719
Treatment Unin	2.238	3.44	0.651	0.5166
Sex Male	-4.748	3.664	-1.296	0.1976
Sex Male*Treatment PBS	9.303	5.446	1.708	0.0903
Sex Male*Treatment Unin	2.838	5.223	0.543	0.5879

Figure S4. Reproductive success during *E. coli* infection. (A) proportion of flies from each treatment that successfully mated. Blue shaded area represents the total number of flies put into mating assay;black bars show the number of flies that produced at least one (1) adult offspring. **(B)** Number of adult offspring resulting from 10/12h mating assays. Large markers indicate means while smaller circles represent individual assays. Bars indicate SE. Experiments were performed at least twice, n= 8-15 biological replicates each. Output from GLM models are shown.

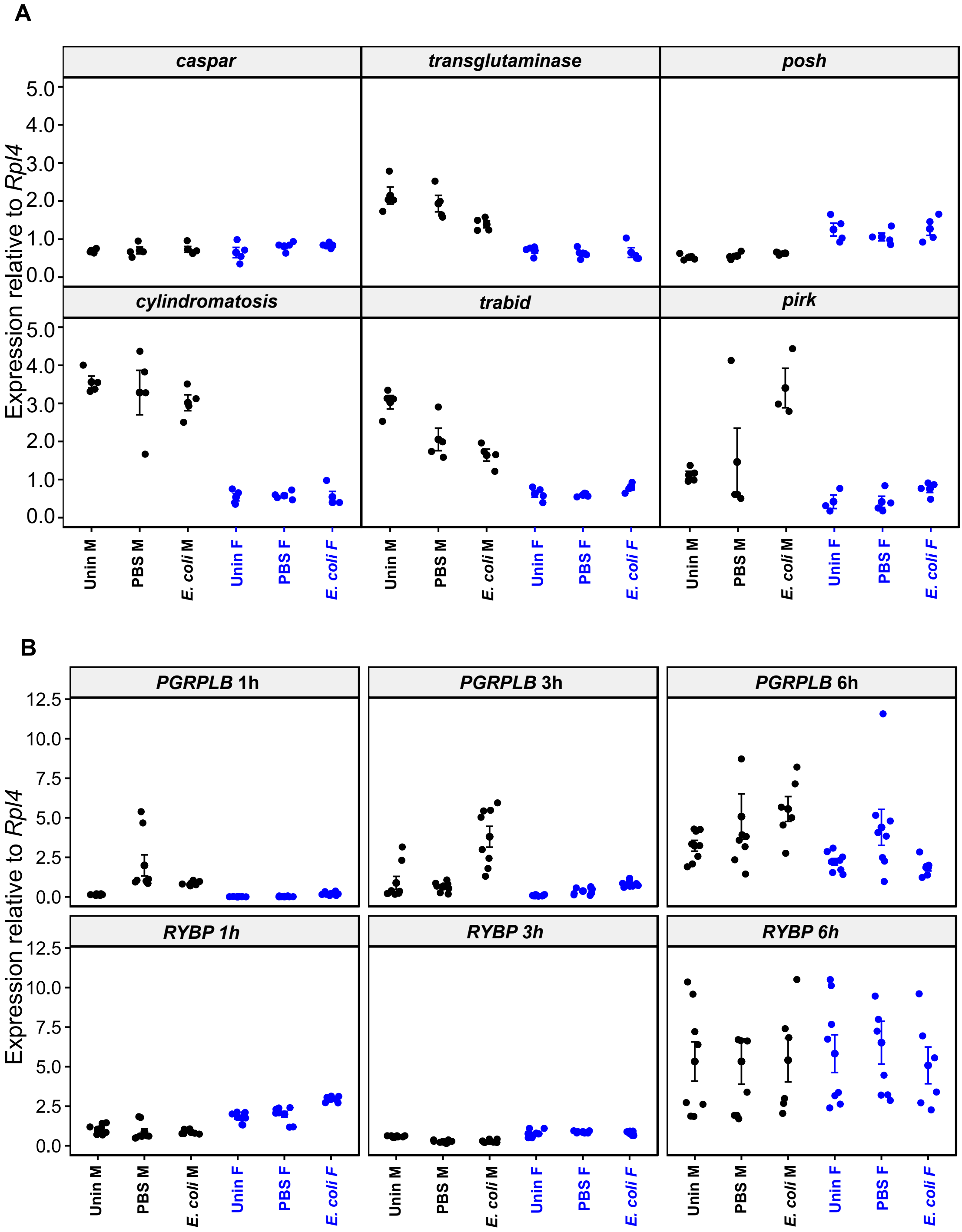

Figure S5. Regulators of the Imd pathway. (A) Expression of known Imd pathway regulators 6h following *E. coli* injection and **(B)** a time course of regulators *PGRP-LB* and *RYBP*. Genes were standardized to the housekeeping gene *ribosomal protein 1*. Data are presented in arbitrary units. Markers represent individual data points. Bars indicate SE. We used 3 or 4 biological replicates/gene, consisting of 3 flies. Time course performed twice.

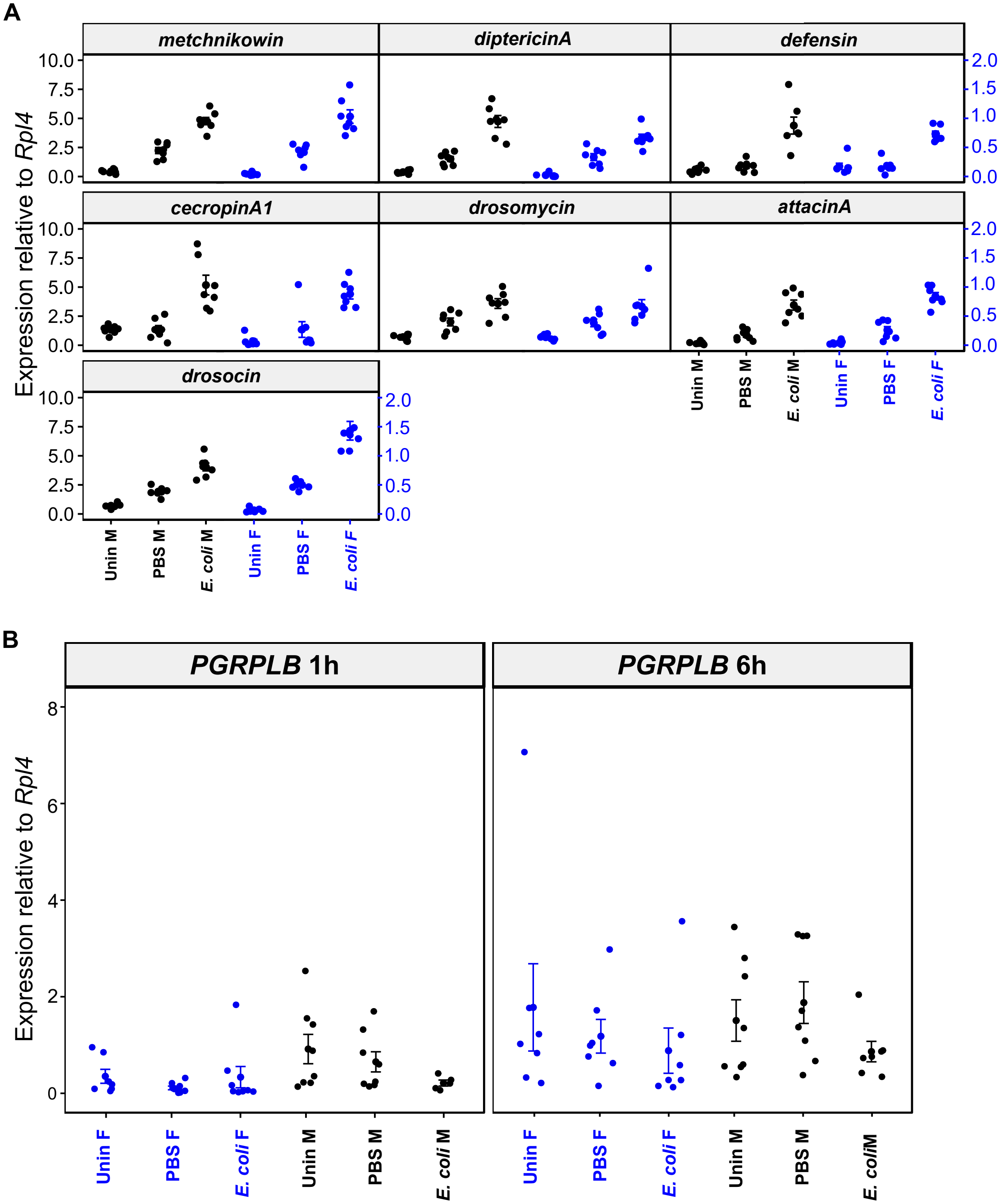

Figure S6. mRNA transcripts following *E. coli* injection.

A. Antimicrobial peptides in *PGRP-LB*Δ mutants 6h after infection.

B.*PGRP-LB* expression in *imd* mutants 1h and 6h after infection.

All genes were standardized to the housekeeping gene *ribosomal protein 1*. Data are presented in arbitrary units. Markers represent individual data points. Bars indicate SE. All assays were performed twice, each repeat included 3-4 biological replicates/treatment consisting of 3 flies each.

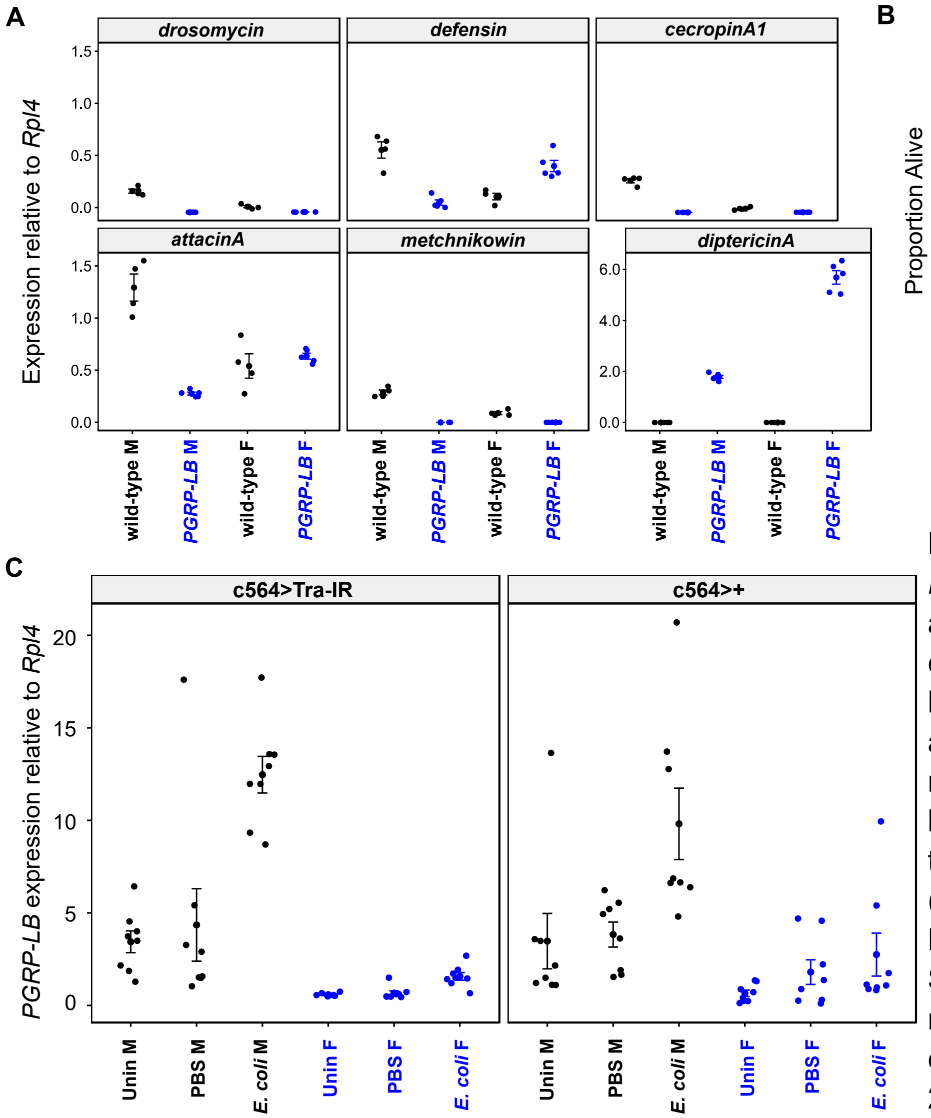

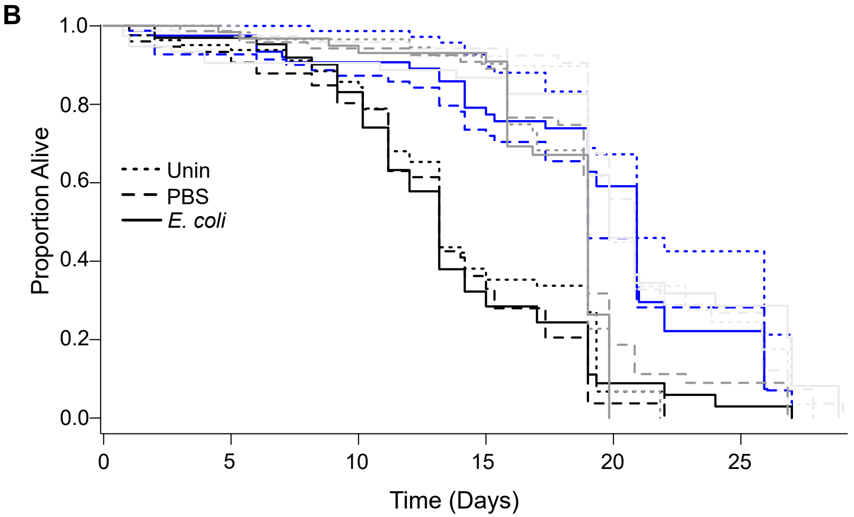

Figure S7. Molecular and survival interactions of *PGRP-LB*. A. Antimicrobial peptide expression of wild-type and *PGRP-LB*Δ flies. **B.** *PGRP-LB* survival with wild-type controls. *PGRP-LB* females and male are represented by blue and black tracings, respectively. Wild-type females and males are represented by light and dark grey tracings, respectively. Infection treatments are as indicated in legend (inset). **C.** Expression of *PGRP-LB* in fat body transformer knock-down (*c564>Trai-IR*) and driver only (*c564>+*) flies. Genes were standardized to the gene *Rpl4*. Markers represent individual data points. Bars indicate SE. All expression assays were performed twice, each repeat included 2-4 biological replicates/treatment consisting of 3 flies each. Survival assays consisted of 20-30 flies/treatment/replicate.

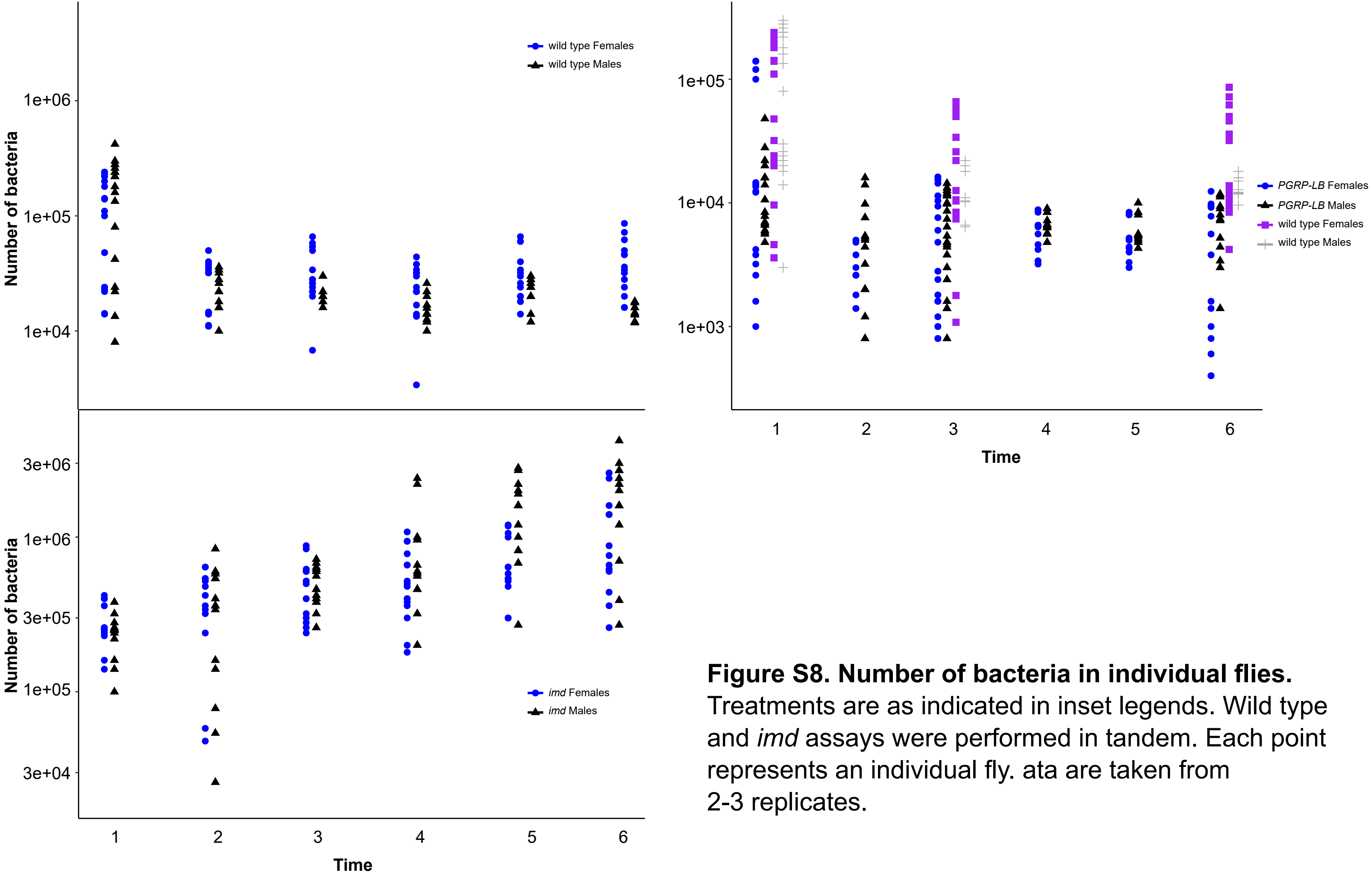

Figure S8. Number of bacteria in individual flies. Treatments are as indicated in inset legends. Wild type and *imd* assays were performed in tandem. Each point represents an individual fly. ata are taken from 2-3 replicates.

Supplementary table 1: wild-type median survival (days)

Treatment	Median survival	upper CI	lower CI	% change
Uninfected M	18.8	19.8	15.8	-
PBS M	19.8	20.8	18.8	+5.3
HK *E. coli* M	19.8	20.8	15.8	+5.3
E. coli M	16.8	NA	15.8	-11.7
Uninfected F	22.8	26.8	19.8	-
PBS F	20.8	25.8	20.8	-9.6
HK *E. coli F*	20.8	20.8	19.8	-9.6
E. coli F	20.8	NA	19.8	-9.6

*HK - heat-killed; M - males; F - females;

% change from uninfected control

Supplementary table 2: Metabolic statistics

wild-type Glucose + Trehalose

Variable	Df	Sum Sq	F	p
Sex	1	0.1404	14.788	0.00027*
Infection status	2	0.0002	0.013	0.98729
Sex*Infection status	2	0.0071	0.371	0.69126

wild-type Glycogen

Variable	Df	Sum Sq	F	p
Sex	1	0.2469	6.153	0.016*
Infection status	2	0.0658	0.82	0.4453
Sex*Infection status	2	0.2209	2.752	0.0721

wild-type Triglyceride

Variable	Df	Sum Sq	F	p
Sex	1	0.0272	1.396	0.2412
Infection status	2	0.2236	5.731	0.00488*
Sex*Infection status	2	0.0182	0.465	0.6296

***imd* Glucose + Trehalose**

Variable	Df	Sum Sq	F	p
Sex	1	0.327	4.302	0.0512
Infection status	1	0.0049	0.065	0.801
Sex*Infection status	1	0.0021	0.028	0.869

***imd* Glycogen**

Variable	Df	Sum Sq	F	p
Sex	1	0.004204	0.22	0.644
Infection status	1	0.004572	0.239	0.630
Sex*Infection status	1	0.015096	0.79	0.385

***imd* Triglyceride**

Variable	Df	Sum Sq	F	p
Sex	1	0	0	0.999
Infection status	1	0.3487	44.971	2.8e-08*
Sex*Infection status	1	0.0575	7.417	9.2e-03*

Supplementary table 3: Metabolic statistics

PGRP-LB Glucose

Variable	Df	Sum Sq	F	p
Sex	1	0.0369	2.576	0.12
Infection status	1	0.0921	6.438	0.017*
Sex*Infection status	1	0.0001	0.007	0.935

PGRP-LB Glycogen

Variable	Df	Sum Sq	F	p
Sex	1	0.0334	0.509	0.4813
Infection status	1	0.6176	9.412	4.8e-03*
Sex*Infection status	1	0.0004	0.007	0.93468

PGRP-LB Triglyceride

Variable	Df	Sum Sq	F	p
Sex	1	0.007	0.499	0.487
Infection status	1	0.0039	0.275	0.605
Sex*Infection status	1	0.0021	0.151	0.701

Supplementary table 4: *PGRP-LB* median survival (days)

Treatment	Median survival	upper CI	lower CI	% change
Uninfected M	13.2	13.2	13.2	-
PBS M	13.2	13.2	11.2	0
E. coli M	11.2	13.2	10.2	-17.9
Uninfected F	25.9	25.9	20.9	-
PBS F	20.9	20.9	17.3	-23.9
E. coli F	20.9	20.9	20.9	-23.9

*M - males; F - females; % change from uninfected control

Supplementary table 5: *PGRP-LB* expression 3h post infection

Treatment	Mean mRNA transcripts (a.u.)	Relative to uninfected
c564> tra-IR		
Uninfected M	3.44	-
PBS M	4.35	1.16
E. coli M	12.47	3.62
Uninfected F	0.58	
PBS F	0.66	1.15
E. coli F	1.57	2.71
c564> +		
Uninfected M	3.47	-
PBS M	3.83	1.36
E. coli M	9.81	3.68
Uninfected F	0.66	-
PBS F	1.79	2.28
E. coli F	2.74	4.03

*M - males; F - females; a.u. arbitrary units
